# Relationship Between Asymptomatic Helicobacter Pylori Infection and BMI Among Patients Undergoing Bariatric Surgery in Saudi Arabia

**DOI:** 10.7759/cureus.37409

**Published:** 2023-04-10

**Authors:** Bandar Saad Assakran, Khaled Alahmadi, Mohammed Alresheedi, Ibrahim Algosair, Hamad Alsuwaidan, Mohammad Harisi, Abdulkarim Alanazi, Abdullah Homood Alromaih

**Affiliations:** 1 General Surgery, King Fahad Specialist Hospital-Buraidah, Buraidah, SAU; 2 Medical School, Qassim University, Buraidah, SAU; 3 Internal Medicine, Qassim University, Buraidah, SAU; 4 Medical School, Qassim University, Riyadh, SAU; 5 Medical School, King Saud Bin Abdulaziz University for Health Sciences, Riyadh, SAU; 6 General Surgery, King Khalid Hospital, Najran, SAU

**Keywords:** endoscopy, overweight, body mass index, helicobacter pylori, obesity

## Abstract

Background

Obesity has become a major health concern associated with several comorbidities. Obesity has been connected to numerous variables. Furthermore, multiple studies were done worldwide to identify the relationship between obesity and Helicobacter pylori (H. pylori), and there was controversy. However, the relationship between H. pylori infection and obesity in our community is still not clear, and there is a knowledge gap.

Aim

To determine the relationship between asymptomatic H. pylori infection and body mass index (BMI) among patients who underwent bariatric surgery in Saudi Arabia, King Fahad Specialist Hospital - Buraidah (KFSH-B).

Method

An observational retrospective cohort study was conducted at KFSH-B. Patients with high BMI (>30 kg/m2) who underwent bariatric surgery between January 2017 and December 2019 were included. Gender, age, BMI, and upper GI endoscopy reports of preoperative mapping were collected from electronic health records.

Results

The sample size was 718, and the mean BMI (standard deviation) was 45 kg (6.8). Patients with positive H. pylori results were 245 (34.1%) and patients with negative H. pylori results were 473 (65.9%). The t-test showed the mean BMI of patients with negative H. pylori results to be 45.36 (SD 6.6). Positive H. pylori 44.95 (SD 7.2) p-value was not significant (0.44).

Conclusion

The data showed that patients who had undergone bariatric surgery had negative pre-operative histopathological results of H. pylori more than those who had positive results, which is consistent with the prevalence of H. pylori infection among the general population. Therefore, we found no correlation between H. pylori infection and high BMI.

## Introduction

Obesity has become a major health concern and became more prevalent over the last hundred years. In Western countries, people who have increased weight are categorized as overweight (BMI of 25-30) and obese (BMI >30). In 2013, the American Medical Association noted that obesity is a disease on its own, not only a risk factor for other disorders, as it reduces the duration of life and alters the activity of many organ systems in the body, and is correlated to the increase of mortality with other comorbidities as diabetes and gastrointestinal diseases. According to the World Health Organization, individuals suffering from obesity are 500 million approximately, which is 10-14% of the world’s population [1,2].

Helicobacter pylori (H. pylori) is a Gram-negative spiral-shaped bacterium that grows in the human stomach [3]. Surprisingly, it has been hypothesized that it correlates with obesity [4]. Additionally, the prevalence of H. pylori information varies but is still high in most countries worldwide, especially in developing countries where it is estimated to be around 80% [3,5,6]. Besides, data available about the prevalence of H. pylori infection in Saudi Arabia is still insufficient but available data shows prevalence in Saudi Arabia ranging from 33% to 85% [7]. H. pylori is known to be a carcinogenic agent causing gastric cancer [3]. Among the infected population, up to 3% progress to gastric cancer [6]. Furthermore, several risk factors are known to be associated with an increased risk of infection, but the most important risk factor is low socioeconomic status while gender and age have no significant effect [3,5].

There is no provided clear indication for preoperative screening and management of H. pylori infection from the American Society for Metabolic and Bariatric Surgery but they do recommend screening in high-prevalence areas with H. pylori and upper endoscopy in selected patients. There is limited data about the prevalence of H. pylori infection in patients with obesity in Saudi Arabia, specifically in the Qassim region, and it is worth mentioning that most of the results that have been illustrated previously were obtained from studies done in developed countries.

Our study was conducted in the Qassim region at King Fahad Specialist Hospital - Buraidah (KFSH-B) in Saudi Arabia which is considered to be a developing country. This may give our study the advantage of an increased possibility of getting varying results. Our aim is to determine the relationship between asymptomatic H. pylori infection and BMI among patients who went for bariatric surgery.

## Materials and methods

This is an observational retrospective cohort study that was conducted at KFSH-B, Qassim, Saudi Arabia. The data were extracted from the medical records and electronic charts of all obese patients who had upper gastrointestinal endoscopy screening between January 2017 - December 2019. Patients with a high body mass index (BMI) >30 kg/m2 were included in our study. Patients with complaints of dyspeptic symptoms including abdominal discomfort or pain or known H. pylori infection, or with symptoms of H. pylori, or who are in treatment with a proton pump inhibitor and with a history of eradication of H. pylori infection were excluded.

Demographic, clinical, and endoscopic data were collected from electronic health records such as gender of participants, age in mean and standard deviation of age group, body mass index, as well as upper GI endoscopy biopsy reports of H. pylori preoperative mapping from the endoscopy unit in our hospital.

Qualitative data such as gender and biopsy results were expressed as frequencies and percentages. Quantitative data such as age and body mass index were expressed as mean and standard deviation. The relationship between asymptomatic H. pylori infection among patients who have undergone bariatric surgery was established using a chi-square test. A non-parametric test was used for non-normally distributed variables, and the variables were expressed as the median and interquartile range. A p-value of <0.05 was considered statistically significant. All statistical analyses were performed using Statistical Packages for Software Sciences (SPSS) version 21 (IBM Corp., Armonk, NY).

The study was approved by the regional research ethics committee, registered at the national committee of Bio & Med. Ethics (NCBE) registration No.H-04-Q-001 under number 1441-2044335.

## Results

In this institution, 718 patients were referred for upper gastrointestinal endoscopy for evaluation before bariatric surgery. There were more female patients, 406 (56.6%) than males, 312 (43.4%). The mean age of those patients was 34 years old and with a standard deviation of ±11.1. The mean BMI of the patients was 45 kg and had a standard deviation of ±6.8. The majority of patients had negative histopathological results of H. pylori infection (473 patients) representing 65.9% of the sample size and the minority had positive histopathological results of H. pylori infection (245 patients) representing 34.1% of the sample size (Table 1).

**Table 1:** Demographic characteristics of patients; n=718 n: number of patients

Variable	
Gender	
Male; n(%)	312(43.4)
Female; n(%)	406(56.6%)
Age; Mean(±SD)	34(11.1)
BMI in Kg; Mean(±SD)	45(6.8)
H. pylori results; n(%)	
Positive	245(34.1)
Negative	473(65.9)

We used the independent sample t-test to identify the correlation between H. pylori infection and the mean BMI of patients. In patients with negative H. pylori results, the mean BMI was 45.36 kg with a standard deviation of ±6.6. On the other hand, the patients with positive H. pylori results showed a mean BMI of 44.95 kg with a standard deviation of ±7.2. These results showing no difference in the mean BMI were confirmed with a t-test which showed the p-value to be 0.440, thus indicating the absence of a correlation (Table 2). 

**Table 2:** T-test

	Mean BMI (±SD)	P VALUE 0.440
Negative H. Pylori results	45.36 (±6.6)	
Positive H. Pylori results	44.95 (±7.2)	

When the t-test showed no correlation, we converted the BMI from continuous data to three categorical groups. We set the BMI of the first group from 30-35 (20 out of 718 patients), and the BMI of the second group was set from 35-40 (188 out of 718 patients). Finally, the last group was set from 40 or more and had 501 out of 718 patients. Around 16 out of 29 patients in the first group showed negative results of H. pylori infection, and the other 13 showed positive results of H. pylori. Regarding patients from the second group, 122 out of 188 patients tested negative for H. pylori infection, and the other 66 tested positive. Lastly, the majority of patients tested positive (335 patients) and 166 with negative results. However, there was no correlation between the BMI of the patients and the risk of H. pylori infection as shown in the chi-square test; the p-value was 0.881 which indicates no correlation (Table 3). 

**Table 3:** Chi-square test to confirm the relationship between BMI and H. pylori infection

Variable	BMI of 30-35	BMI of 35-40	BMI of 40 or more	P-value 0.881
Negative H. pylori results	16	122	335	
Positive H. pylori results	13	66	166	

We wanted to see if there is any correlation between H. pylori results and gender. The total female number was 406, 270 out of them had negative H. pylori results and 136 had positive results. There were a total of 312 males; 203 of them had negative results of H. pylori infection, and 109 patients had positive H. pylori infection. The p-value was 0.861 which indicates that there is no correlation between gender and H. pylori infection results (Table 4). 

**Table 4:** Correlation between H. pylori results and gender

Variables	Male	Female	p-value 0.861
Negative H. pylori result	203	270	
Positive H. pylori result	109	136	

## Discussion

The prevalence rates of H. pylori infection vary among countries, with increased rates in developing countries up to 80-90% [8]. Likewise, a number of community-based studies were conducted on the healthy Saudi population from several regions in the kingdom, and most of these studies were among adults, reporting a prevalence rate that ranged between 23-67% [9-11]. Due to the lack of collected data for H. pylori among the Saudi population, one of the aims of this study is to find the distribution pattern regarding demographic characteristics, the prevalence of asymptomatic H. pylori patients, and its correlation with high BMI before undergoing bariatric surgery.

In regard to gender groups with respect to positive H. pylori infection, the study revealed that 34.9% of males and 34.5% of female patients had positive results, which to some extent, is not worth considering seeing the minor difference between both genders. Similarly, a case-control study adopted at a central hospital (King Khalid) in the Najran region southwest of Saudi Arabia showed non-significant findings, 146 (64.6%) in males versus 79 (69.3%) in females. By observing the above results, we can exclude the linkage of H. pylori infection to both genders [7].

The mean age of our study was 34, which is low in comparison to a study done in 2016 in Prince of Wales Hospital, Sha Tin Hong Kong, China, which showed a mean age of 39.1 years. Furthermore, the mean BMI in our study was 45(±6.8), which is in accord with the previously mentioned study where the BMI was 40.3, which is considered morbidly obese [12]. Our study shows that H. pylori were positive in 245 patients accounting for 34.1% of the total investigated patients, which was considered low when compared to a study collected in 2018 in Pakistan where the result of H. pylori infection was detected in 399 out of 698 patients representing (57%) [12]. In contrast, the prevalence of H. pylori infection in our study is considered high when compared to a similar British study that has shown positive results of H. pylori in 1636 of a total of 10537 patients, representing only 16% of the cohort. This significant disparity in these outcomes is consistent with other similar studies that reported a high prevalence of H. pylori infection in developing countries compared to developed countries.

In our study, we tried to find a correlation between high BMI and the increased risk of H. pylori infection, so we used a t-test. As a result, our study revealed that in patients with negative H. pylori results, the mean BMI was 45.36 kg, while the patients with positive H. pylori results showed a mean BMI of 44.95 kg. With the aforementioned results that show a p-value of 0.440, we inferred that there is no correlation between high BMI and H. pylori infection.

Moreover, we subdivided BMI into three groups to find a correlation between high BMI and increased risk of H. pylori using the chi-square test. Consequently, the BMI of the first group of patients ranged from 30-35 kg, and they were 29/718 patients with a prevalence of H. pylori at 44.8%. The second group's BMI fell between 35-40, accounting for 188/718, with the prevalence of patients having H. pylori at 35.1%. Furthermore, the third group's BMI was more than 40, accounting for 501/718, with the prevalence of H. pylori at 33.1%. In conclusion, we found a higher prevalence of H. pylori in the first two groups falling between 30-40 BMI, compared to subjects that fell in 40 or more. Finally, the chi-square test showed a p-value of 0.881 which denotes the absence of correlation between high BMI groups and the risk of H. pylori infection.

We faced many issues during data collection, the most important one is that some variables were missed in the system.

## Conclusions

The aim of the article was to find the relationship between H. pylori infection and high BMI in patients who had undergone bariatric surgery. However, we found no relation between them. We distributed patients into three categories according to their BMI 30-35, 35-40, and 40 or more. Similar outcomes were discovered. Then, we tried to find an association between the two by gender and found no association. 

Data showed that patients who had undergone bariatric surgery had negative pre-operative histopathological results of H. pylori more than those who had positive results, which is consistent with the prevalence of H. pylori infection among the general population. Therefore, there is no correlation between H. pylori infection and high BMI.

## References

[1] Lender N, Talley NJ, Enck P, Haag S, Zipfel S, Morrison M, Holtmann GJ (2014). Review article: Associations between Helicobacter pylori and obesity--an ecological study. Aliment Pharmacol Ther.

[2] Chen J, Ma J, Liu X, Duan S, Liang N, Yao S (2020). The association between Helicobacter pylori infection with overweight/obesity: A protocol for a systematic review and meta-analysis of observational studies. Medicine (Baltimore).

[3] Kotilea K, Bontems P, Touati E (2019). Epidemiology, Diagnosis and Risk Factors of Helicobacter pylori Infection. Adv Exp Med Biol.

[4] Cho I, Blaser MJ, François F, Mathew JP, Ye XY, Goldberg JD, Bini EJ (2005). Helicobacter pylori and overweight status in the United States: data from the Third National Health and Nutrition Examination Survey. Am J Epidemiol.

[5] Eusebi LH, Zagari RM, Bazzoli F (2014). Epidemiology of Helicobacter pylori infection. Helicobacter.

[6] Wang F, Meng W, Wang B, Qiao L (2014). Helicobacter pylori-induced gastric inflammation and gastric cancer. Cancer Lett.

[7] Al-Zubaidi AM, Alzobydi AH, Alsareii SA, Al-Shahrani A, Alzaman N, Kassim S (2018). Body Mass Index and Helicobacter pylori among Obese and Non-Obese Patients in Najran, Saudi Arabia: A Case-Control Study. Int J Environ Res Public Health.

[8] Bruce MG, Maaroos HI (2008). Epidemiology of Helicobacter pylori infection. Helicobacter.

[9] al-Moagel MA, Evans DG, Abdulghani ME, Adam E, Evans DJ Jr, Malaty HM, Graham DY (1990). Prevalence of Helicobacter (formerly Campylobacter) pylori infection in Saudia Arabia, and comparison of those with and without upper gastrointestinal symptoms. Am J Gastroenterol.

[10] Hanafi MI, Mohamed AM (2013). Helicobacter pylori infection: seroprevalence and predictors among healthy individuals in Al Madinah, Saudi Arabia. J Egypt Public Health Assoc.

[11] Habib HS, Hegazi MA, Murad HA, Amir EM, Halawa TF, El-Deek BS (2014). Unique features and risk factors of Helicobacter pylori infection at the main children's intermediate school in Rabigh, Saudi Arabia. Indian J Gastroenterol.

[12] Lee J, Wong SK, Liu SY, Ng EK (2017). Is Preoperative Upper Gastrointestinal Endoscopy in Obese Patients Undergoing Bariatric Surgery Mandatory? An Asian Perspective. Obes Surg.

